# Training discrimination diminishes maladaptive avoidance of innocuous stimuli in a fear conditioning paradigm

**DOI:** 10.1371/journal.pone.0184485

**Published:** 2017-10-12

**Authors:** Miriam J. J. Lommen, Mihaela Duta, Koen Vanbrabant, Rachel de Jong, Keno Juechems, Anke Ehlers

**Affiliations:** 1 Department of Experimental Psychology, University of Oxford, Oxford, United Kingdom; 2 Oxford Cognitive Health NIHR Clinical Research Facility, University of Oxford, Oxford, United Kingdom; 3 Center for the Psychology of Learning and Experimental Psychopathology, University of Leuven, Leuven, Belgium; Swansea University, UNITED KINGDOM

## Abstract

Anxiety disorders are the most common mental disorder worldwide. Although anxiety disorders differ in the nature of feared objects or situations, they share a common mechanism by which fear generalizes to related but innocuous objects, eliciting avoidance of objects and situations that pose no objective risk. This overgeneralization appears to be a crucial mechanism in the persistence of anxiety psychopathology. In this study we test whether an intervention that promotes discrimination learning reduces generalization of fear, in particular, harm expectancy and avoidance compared to an irrelevant (control) training. Healthy participants (N = 80) were randomly allocated to a training condition. Using a fear conditioning paradigm, participants first learned visual danger and safety signals (set 1). Baseline level of stimulus generalization was tested with ambiguous stimuli on a spectrum between the danger and safety signals. There were no differences between the training groups. Participants then received the stimulus discrimination training or a control training. After training, participants learned a new set of danger and safety signals (set 2), and the level of harm expectancy generalization and behavioural avoidance of ambiguous stimuli was tested. Although the training groups did not differ in fear generalization on a cognitive level (harm expectancy), the results showed a different pattern of avoidance of ambiguous stimuli, with the discrimination training group showing less avoidance of stimuli that resembled the safety signals. These results support the potential of interventions that promote discrimination learning in the treatment of anxiety disorders.

## Introduction

Anxiety disorders are the most common mental disorders [1,2] and are characterized by excessive fear and avoidance. While the type of feared stimuli varies across the different anxiety disorders, e.g., social interactions in social anxiety disorder, spiders is spider phobia, intense exercise in panic disorder, a common feature is that the patients' fear has generalized to innocuous stimuli or situations and elicits excessive avoidance responses. The fear and avoidance can dominate patients' lives by impairing physical, social, emotional, and professional functioning [3].

There is a growing interest in the mechanisms of fear generalization. Fear conditioning paradigms have been used to investigate this process in the laboratory. After a conditioning phase, these paradigms measure responses to ambiguous or generalized stimuli (GS) that share features of learned danger (CS+) and safety (CS-) signals to varying degrees. The subjective reaction (e.g., harm expectancy) and the physiological activity these GSs evoke are usually assessed as indicators of the level of fear generalization. Fear generalization is operationalized as the transference of the fear evoked by the danger signal to stimuli that are either perceptually similar to the CS+, or non-perceptually related to the CS+, for example by being semantically or symbolically related to the CS+ (for an overview see [4]).

Compared to healthy controls, patients with generalized anxiety disorder [5] or panic disorder [6], tend to show an overgeneralization of fear: they tend to generalize their fear response to stimuli that are less closely related to the CS+, and to a larger number of stimuli in general. One of the ways the overgeneralization is assessed in clinical samples is the shape of the curve when the stimuli (CS-, GSs and CS+) are plotted on the x-axis and the responses to the CSs on the Y-axis: the curve is more quadratic in the healthy control group and more linear in the anxiety patient group (e.g., [5]), reflecting increased responses towards GSs in the anxiety population. Although not all studies have found this overgeneralization effect [7,8], there is substantial evidence for the role of fear generalization in anxiety pathology [4,9]. An important limitation of the research in fear generalization is the scarcity with which overt behaviour or avoidance tendencies have been included as an index of fear [10,11]. Avoidance behaviour in response to innocuous stimuli constitutes one of the major aspects of diagnostic criteria for anxiety disorders [12]. Moreover, avoidance seems to play a crucial role in the maintenance and possibly even exacerbation of pathological anxiety by preventing disconfirmation of irrational or exaggerated danger beliefs[13]. Thus, assessing avoidance behaviour in fear generalization paradigms seems to have important clinical relevance. Thus far, only a few studies have done this by studying learning and generalization of avoidance [14,15], showing support for an association between overgeneralization of fear and overgeneralization of avoidance [11], and enhanced avoidance of GS in anxiety prone individuals (i.e., high neuroticism, [16]).

A next challenge in the field of fear generalization is to further insight into interventions that target (over)generalization of fear and avoidance. A first successful attempt has recently been shown in a study in healthy adults. Compared to a control group, those who were trained to spot small differences in contour and size of stimuli (stimulus discrimination) reported increased risk ratings of generalized stimuli of which the colour was lying on a continuum between the colour of CS+ and CS-. Although these differences in fear generalization were not present in the fear potentiated startle responses, the results show that a training promoting perceptual discrimination might be a promising way to reduce fear generalization [17]. Since avoidance responses were not included it remains unclear whether such a training task would target avoidance generalization. In this study we tested whether a training to enhance stimulus discrimination would result in less overgeneralization of fear and avoidance compared to an irrelevant (control) training.

The training used in this study aimed at diminishing generalization of fear and avoidance was inspired by a clinical intervention “stimulus discrimination”, which is part of the cognitive therapy for posttraumatic stress disorder (PTSD) [18,19]. In the clinical intervention, PTSD patients learn to identify triggers of their intrusive memories (often subtle sensory cues) and then learn to discriminate these by focusing their attention on the differences between the triggers and their current context from those in the actual trauma. For example, a road traffic accident survivor who survived a head-on car crash at night identified that bright light on a dark background triggered his intrusions. In therapy, he looked at different kinds of bright lights against dark backgrounds, while focusing on differences in the perceptual pattern and the context in which he observed the light. He learned to discriminate the lights and their present context from the headlights he saw in the darkness of the night before his accident [19]. Similar to this clinical intervention, the training in this study aimed to promote discrimination of stimuli with perceptual similarities.

In sum, overgeneralization of fear and avoidance appears to play a major role in the maintenance of anxiety disorders. This study investigates the effects of training that promotes perceptual stimulus discrimination on the generalization of fear, in particular, harm expectancy and avoidance. To rule out that potential differences in fear (over)generalization between the training groups can be attributed to other third factors, this study will test and if needed control for group differences in the acquisition of fear, or individual differences in trait anxiety, neuroticism and worry.

## Methods

### Participants

Via posters and online advertisements, a total of 80 members of the Oxford community, most of them student at the University of Oxford or Oxford Brookes University, were recruited and selected to take part in this study. Exclusion criteria were (1) past or current psychiatric or drug-related disorder, (2) visual problems (unless corrected), (3) colour blindness, (4) use of medication or drug that could interfere with attention, reaction capacity and/or memory, (5) epilepsy, (6) heart condition, (7) current pregnancy, (8) a clinical score or suicidal ideation according to the Beck Depression Inventory (BDI-II), (9) age below 18, and (10) non-fluency in English. Since two participants could not complete the study due to technical difficulties, a total of 78 participants completed the study, with 39 participants in each training group.

### Procedure

The study had been approved by the ethical committee of the University of Oxford and took place in a temperature-controlled, sound-attenuated room. After participants gave written informed consent, exclusion criteria were checked with an interview, followed by the Ishihara Test to check for colour blindness [20]. Using a work-up procedure (i.e., [21]), the intensity of the electric shock that was used as unconditioned stimulus (US) was set to an individual level that was ‘highly annoying but not painful’. Then the conditioning paradigm started. Participants completed questionnaires at the end of the session and received a financial compensation of GBP 15 for their participation.

### Conditioning paradigm

The conditioning paradigm consisted of different phases in which two stimuli sets were used.

#### Stimuli

**Fig 1:**
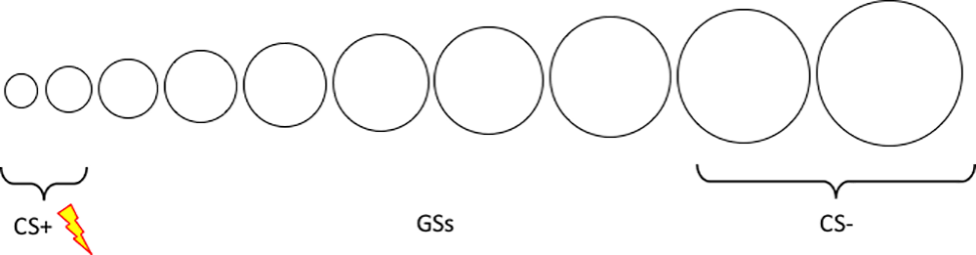
Stimulus set one.

**Fig 2:**
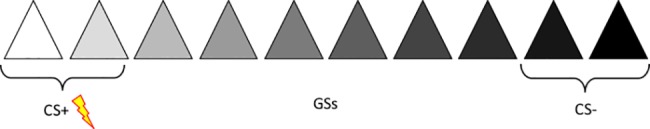
Stimulus set two.

Two sets of 10 stimuli (Fig 1 and Fig 2) were used in the de novo conditioning computer task [21]. The first set of stimuli ranged from small to large sized white circles (i.e., [22]), and was used before the training phase. The diameter for the smallest circle was 1.50 in, and the diameter for the largest circle was 4.20 in. The two smallest circles and two largest circles were used as CS+ and CS-, counterbalanced across participants in each group. The six intermediately sized circles served as generalized stimuli (GSs).

The second set of stimuli included triangles on a spectrum from white to black (i.e., [16]), and was used after the training phase. The diameter for the triangles was 1.85 in. The two lightest and two darkest triangles were used as CS+ or CS-, counterbalanced over the groups. The six degraded grey colours served as generalized stimuli (GSs).

All stimuli were presented for 6 seconds on a computer monitor, with an Inter Trial Interval (ITI) of 9–12 seconds in semi-randomized order. The US was a 500 ms mild electric shock, delivered by the Coulbourn Finger Stimulator via finger electrodes on two fingers of the dominant hand (0.0–4.0 mA).

#### Pretraining acquisition and generalization

Stimulus Set 1 was used for this experimental phase.

**Habituation phase I**. Each CS+ and CS- was presented twice without the US, resulting in a total of 8 trials.

**Acquisition phase I**. Each CS+ and CS- was presented 4 times, resulting in a total of 16 trials. Each CS+ presentation was followed by a US. CS+ and CS- trials were presented in a semi-random order, such that no more than two stimuli of the same class occurred consecutively.

**Generalization phase I**. CS-s and the GSs were presented in a set order, starting with the CS- up to the GS closest to the CS+. None of the CS- or GS presentations was followed by a US.

#### Training phase

Participants were randomly assigned to the relevant or irrelevant training group. The relevant (stimulus discrimination) training consisted of 30 trials in which two different stimuli were presented shortly after each other. The participants’ task was to decide whether the stimuli differed in terms of shape, colour, or not at all while trying to make as few mistakes as possible. In this training phase, the same colours were used as in stimulus set 2. In the generalization phase after the training phase, the shapes used differed from the ones used in stimulus set 1 and 2. Participants received audio feedback on their choice: a buzzer sound (low tone) if the answer was wrong, and a high-pitched bleep sound (high tone) if the answer was right. Both sounds were introduced to participants before the start of the phase to prevent any misunderstanding about their meaning. This relevant training aimed to promote stimulus discrimination by asking the participants to focus on subtle differences in physical resemblance of the stimuli.

In the irrelevant (control) training, the same set of stimuli was used as in the relevant training group, however, participants were presented one stimulus at the time and had to decide as quickly as possible whether the word in the left or the right bottom corner of the screen related to the stimulus. The words related to the shape (e.g. circle or trapezium) or the colour (e.g. milk or elephant) of the stimulus. The training was presented as a reaction time task, and participants were instructed to respond as quickly as possible while making as few mistakes as possible. They received audio feedback on their choice and their reaction time was shown. The irrelevant training did not promote stimulus discrimination as it did not ask the participant to focus on the subtle differences in physical resemblance of the stimuli, but on their semantic knowledge related to the stimuli.

#### Posttraining acquisition and generalization

Stimulus Set 2 was used for this experimental phase.

**Habituation phase II**. Each CS+ and CS- was presented twice without the US, resulting in a total of 8 trials.

**Acquisition phase II**. Each CS+ and CS- was presented 4 times, resulting in a total of 16 trials. Each CS+ presentation was followed by a US. CS+ and CS- trials were each presented in a semi-random order, such that no more than two stimuli of the same class occurred consecutively. Additionally, the function of the lights was explained: Participants were instructed that a steady yellow or a flickering white light would be shown before stimulus onset. The flashing white light indicated that the participant could avoid a potential US by pressing the space bar within four seconds after CS onset. All 16 trials were preceded by the yellow light.

**Generalization phase II**. In this phase the CS-s and GSs were each presented twice semi-random order, such that no more than two stimuli of the same class occurred consecutively, and were all preceded by the white flickering light to allow avoidance responses. Since none of the CS-s or GSs were actually followed by a US, the CS+s were each presented twice at set points with the yellow light, followed by the US to prevent extinction learning (i.e., [16]). CS+s were never presented with the white flickering light as reinforcing unavoided CS+s would lead to a difference in US exposure and thus make the participants incomparable, while not reinforcing the CS+ would elicit early extinction learning for those who do not avoid CS+s.

#### Extinction and ratings

**Extinction phase**. To invalidate the CS/US contingency, CS+s and CS-s (stimulus set two) were each presented five times without the US.

At the end of the conditioning task, participants rated the aversiveness of the US on a 0 (*not unpleasant*) to 100 (*extremely unpleasant*) mm visual analogue scale (VAS).

### Outcome measures

**Cognitive outcome measure (harm expectancy)** During each CS and GS presentation, except for the habituation phases, participants rated their expectancy of the shock (US expectancy) on a 0 (*certainly no shock*) to 100 (*certainly a shock*) mm VAS presented on the computer screen during the first 5 s of the 6 s stimulus presentation.

**Behavioural outcome measure** In the generalization phase II (after the training phase), the number of behavioural responses to avoid a potential US during each CS- and GS presentation was recorded.

### Questionnaires

Depressive symptoms were assessed with the Beck Depression Inventory (BDI-II; [23]). The BDI-II consists of 21 items that are rated on a 4-point Likert scale (0 to 4). The total score and score on question number 9 (suicidal ideation) were used to exclude vulnerable individuals. In this sample, the Cronbach’s alpha was .84.

Trait anxiety was measured with the trait anxiety scale of the State-Trait Anxiety Inventory (STAI-T; [24]). This self-report inventory based on a 4-point Likert scale consists of 20 questions and measures anxiety level as a personality characteristic. The scale ranges from 1 (*not at all*) to 4 (*very much so*). In this sample, the Cronbach’s alpha was .91.

Neuroticism was assessed with the neuroticism scale of the Eysenck Personality Questionnaire (EPQ-N; [25]). This self-report scale consists of 22 items rated as 0 (*no*) or 1 (*yes*). In this sample, the Cronbach’s alpha was .81.

Worry was measured with the Penn-State Worry Questionnaire (PSWQ;[26]). This self-report scale consists of 16 items that are rated on a 5-point Likert scale from 1 (*not at all typical of me*) to 5 (*very typical of me*). In this sample, the Cronbach’s alpha was .91.

## Results

All data collected in this study has multiple observations per participant. To deal with these dependencies in the data we used a hierarchical modeling approach [27]. Use of hierarchical models is a more appropriate way of analyzing generalization data than using repeated measures ANOVA, as the latter treats the generalization stimuli as a categorical dimension whereas a dimensional approach seems more appropriate. Because hierarchical models allow the inclusion of the stimulus dimension as a continuous variable, it is possible to model the strength of the response as a parametric function of the stimulus dimension (i.e., more specifically as a quadratic function). Moreover, the assumption of sphericity in a repeated measures ANOVA, that is equality of variances within stimuli and correlations between stimuli, is not realistic when working with generalization data, resulting in increased probability of type I error. For a full discussion of the benefits of the used approach over a repeated measures ANOVA, see [27]. All models were fitted in R [28] by means of the lme4 package [29].

The main research aim was to test whether the relevant training that promoted perceptual stimulus discrimination diminished the generalization of fear, in particular, harm expectancy and avoidance compared to an irrelevant training. However, first several analyses were run to rule out any group differences in fear acquisition that could account for potential differences in fear generalization.

### Descriptive statistics

Of the 78 participants who completed the study, 24 (30.8%) were male. The relevant and irrelevant training groups did not differ with regard to gender ratio within the group, χ^2^ = .96, *p* = .32. The mean age was 26 years, (*SD* = 5.75, range 18–76) and the groups did not differ in age, *t*(76) = 1.39, *p* = .17. The mean shock level chosen by the participants was 1.9 mA (*SD* = 1.02) and did not differ between the groups, *t*(76) = -0.11, *p* = .91. There were no group differences on the questionnaires assessing trait anxiety (STAI-T), *t*(75) = -0.37, *p* = .71, neuroticism (EPQ-N), *t*(76) = -0.08, *p* = .94, depressive symptoms (BDI-II), *t*(76) = 0.19, *p* = .85, or worry (PSWQ), *t*(76) = 0.18, *p* = .86.

In order to study fear generalization, adequate fear acquisition is a prerequisite. Therefore only participants who correctly learned the CS/US contingency by the end of the acquisition phase (i.e., expectancy of >75 for the last CS+ trial and an expectancy of <25 for the last CS- trial) were included in the data analysis, resulting in the inclusion of 65 participants in the pre-training generalization phase and 66 participants in the post-training generalization phase.

### Acquisition data

The question of interest here is twofold: First, did participants acquire differential reactions to the CS+ and CS- trials in acquisition phase I and II? Second, were there differences in acquisition for participants who later received the two training conditions? Both questions were answered by comparing nested hierarchical models. All models included a varying intercept over all participants to account for the repeated measures nature of the data. The comparison of the nested models was done with a difference in Deviance test. The first model (model A) only included the trials as a within subjects factor. The second model (Model B) included the CS type as a within subject factor and the interaction between trial number and CS type. To test whether participants acquired different US expectancies for CS+ and CS- trials in the acquisition phases, it was tested whether model A and B differed significantly. To test group differences in fear acquisition, a more extended model was introduced (model C). This model included all effects of model B and was expanded with a third order interaction effect of trials, CS type, and training condition (and second order interactions between training condition and the other variables and the main effect of this training condition).

#### Acquisition phase I (pre-training)

The acquisition curve was based on US expectancies of the CS+ and CS- over all trials. Results showed a significant improvement in model fit of Model B over model A, *χ*^*2*^ (8) **=** 1747.80, *p* < .01. This indicates that participants learned to discriminate between the CS+ and CS- trials. A comparison between model B and model C indicates that there were no differences between the two training conditions in their pre-training acquisition, *χ*^*2*^ (16) **=** 15.62, *p* = .48.

#### Acquisition phase II (post-training)

The acquisition curve was based on US expectancies of the CS+ and CS- over all trials. The same tests were run as for the acquisition phase I, which resulted in similar results: a significant difference was found between the CS+ and CS- trials, *χ*^*2*^ (8) = 1891.9, *p* < .01, indicating sufficient acquisition learning. There were no differences between the training conditions in the rate of acquisition of the CS+ and CS- differences, *χ*^*2*^ (16) = 11.149, *p* = .80, which suggests that potential differences between the two training groups in fear generalization are not attributable to differences in the acquisition of the CS+ and CS- differences.

### Generalization data

A different approach was used for the analysis of the generalization data, as the mean structure of the tested model was given by the research question. The model included a varying intercept, again, to control for the repeated measures nature of the data, a linear effect of the stimulus dimension, a quadratic effect of the stimulus dimension, a main effect of the training condition, and a cross-level interaction between the linear stimulus dimension and quadratic stimulus dimension. This later interaction effect is of main interest in this analysis. The stimulus dimension is included as a continuous variable that was mean-centered for computational reasons.

First, this model was expanded with a varying linear stimulus dimension, and second, expanded with a varying quadratic stimulus dimension to test for an optimal random effects structure. The random effects structure always takes the forms of an unstructured variance-covariance matrix. These models were then compared by means of the difference in deviance. Once the final model was established, the parameters of interest were tested using a Wald test.

#### Generalization phase I (pre-training; US-expectancy)

**Fig 3:**
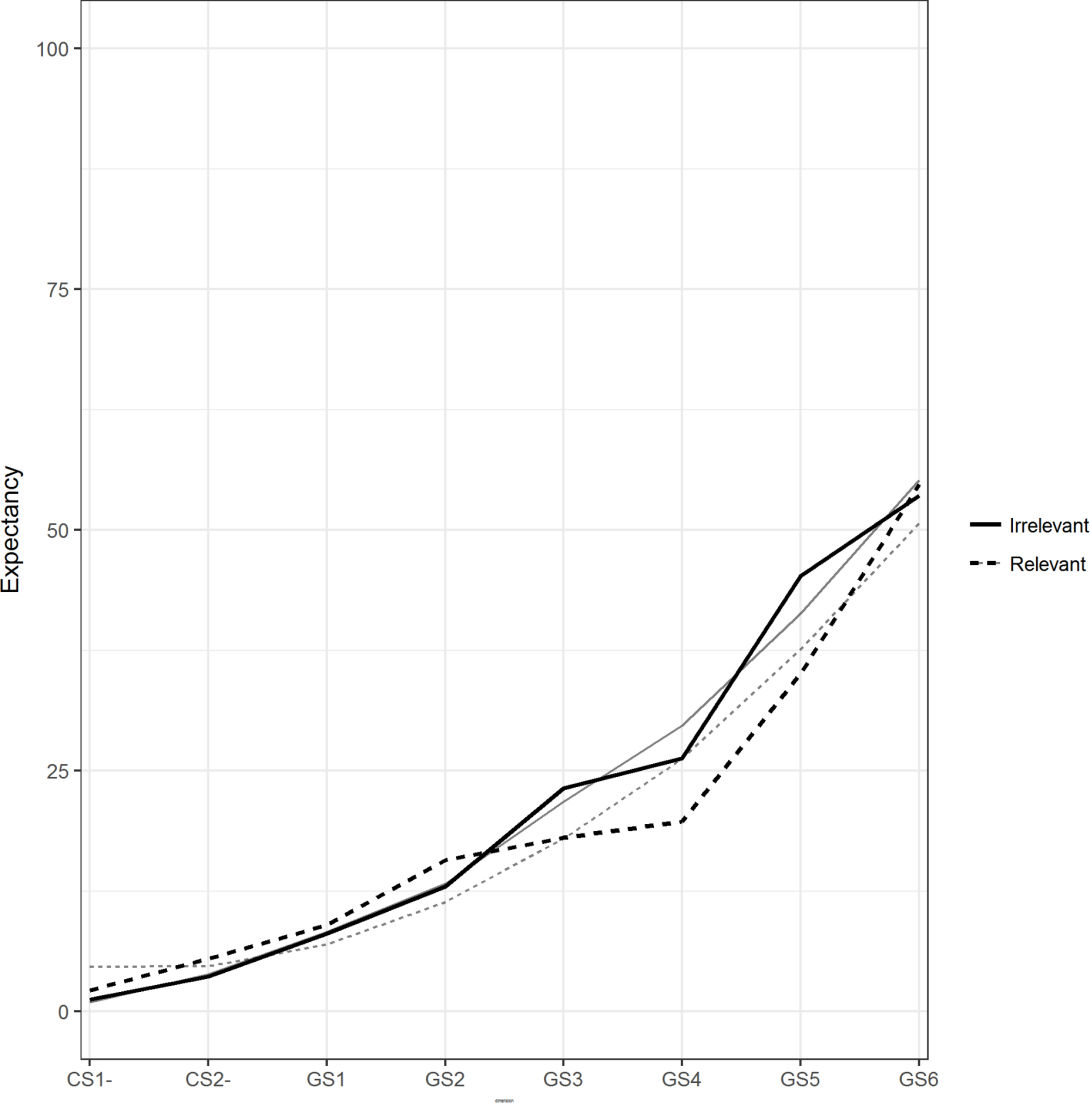
Mean pre-training generalization gradients for both training conditions with US-expectancy as an outcome measure. The black lines represent the mean observed data, the grey lines the mean model predictions per condition, with solid lines for the irrelevant condition and dotted lines for the relevant condition.

US-expectancy ratings were collected for the CS-s and the six GSs. The mean generalization gradients for the two training conditions are visualized in Fig 3. A model with an additional random effect of the stimulus dimension had a better fit compared to the random intercept model, *χ*^*2*^ (2) = 92.19, *p* < .01. An additional random effect of the quadratic stimulus dimension fit the data even better,*χ*^*2*^ (3) = 55.193, *p* < .01, and was used as final model. The parameter estimates for this model are provided in Table 1 (see Model 1). The model shows that the gradients have a significant linear and quadratic slope, but that there are no differences between the two training groups.

**Table 1:** Parameter estimates and standard error (SE).

	Pre Expectancy		Post Expectancy		Post Avoidance	
Intercept	17.6 5**	(3.53)	42.0 0**	(5.13)	-0.00	(0.73)
Dimension	7.4 1**	(0.92)	11.4 4**	(0.44)	1.26**	(0.19)
Training	-3.26	(4.95)	9.25	(7.14)	-1.35	(1.01)
Dimension^2^	0.7 6*	(0.37)	0.44	(0.31)	-0.05	(0.05)
Dimension*Training	-0.83	(1.28)	0.60	(0.61)	0.06	(0.24)
Dimension^2^ *Training	0.33	(0.52)	-0.48	(0.43)	0.1 3*	(0.07)
AIC	4552.84		5933.01		644.53	
Log Likelihood	-2263.42		-2953.50		-313.27	
Num. obs.	512		659		528	
Num. Subjects	65		66		66	
Variance: Intercept	321.05		762.02		13.60	
Variance: dimension	20.24		2.01		0.41	
Variance: dimension^2^	2.80		2.37		-	
Variance: Residual	260.05		345.71		-	

Note. AIC = Akaike Information Criterion, Num. obs. = number of observations, num. Subjects = number of subjects

** *p*<0.001

* *p*<0.05

#### Generalization phase II (post-training; US-expectancy)

**Fig 4:**
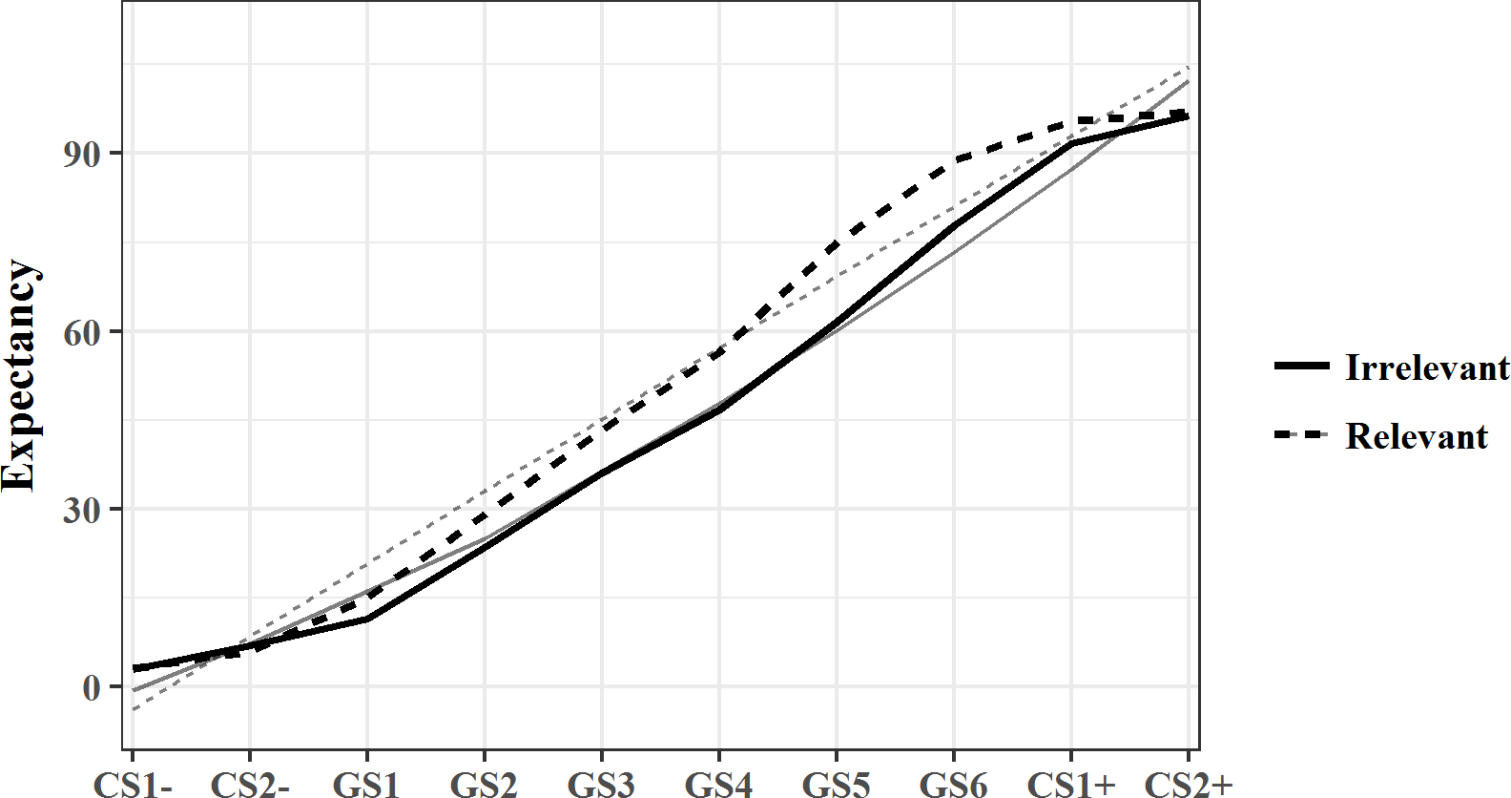
Mean post-training generalization gradients for both training conditions with US-expectancy as an outcome measure. All CSs are represented on the X-axis, with CS-s at the left, GSs in the middle and the CS+s at the right side. The black lines represent the mean observed data, the grey lines the mean model predictions per condition, with solid lines for the irrelevant condition and dotted lines for the relevant condition.

US-expectancy ratings were collected for the CS+s, CS-s and six GSs. The mean generalization gradients for the two training conditions are visualized in Fig 4. A model with an additional random effect of the stimulus dimension did not show a better fit than the random intercept model, *χ*^*2*^ (23) = 0.01, *p* = 1.00, but a model with a quadratic random slope reached a better fit compared to the random intercept model, *χ*^*2*^ (5) = 176.65, *p* < .01. In addition to the random quadratic stimulus dimension, the linear stimulus dimension was also included, as lower order functions need to be included in the model when higher order functions are present [30].

The parameter estimation of the latter model can be found under “post expectancy” in Table 1. The model shows that the gradients have a significant linear and quadratic slope, but that there are no differences between the two training groups.

#### Generalization phase II (post-training; avoidance responses)

**Fig 5:**
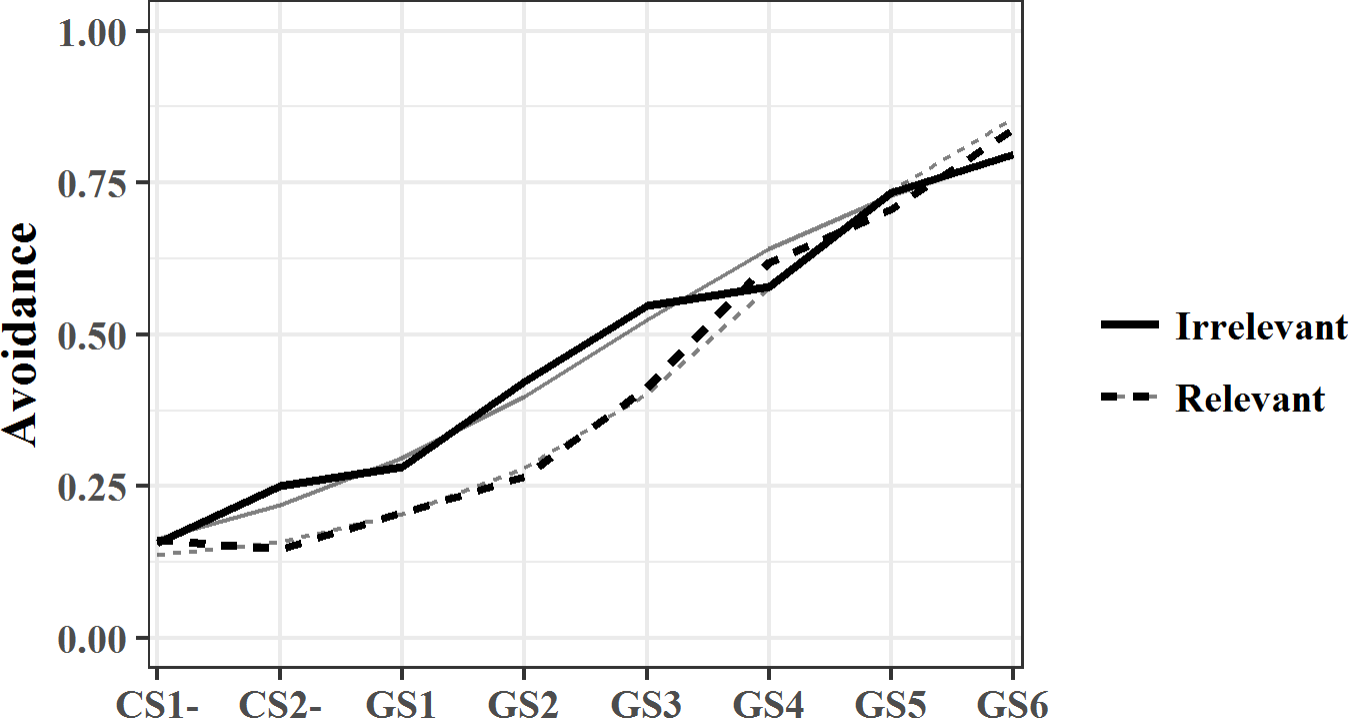
Mean post-training generalization gradient for both training conditions with probability of avoidance as an outcome measure. All avoidable CSs are represented on the X-axis, with CS-s at the left and the GS most similar to CS+ at the right side. The black lines represent the mean observed data, the grey lines the mean model predictions per condition, with solid lines for the irrelevant condition and dotted lines for the relevant condition.

Every avoidable stimulus (CS-s and GSs) was presented twice, which means that each stimulus could be avoided 0, 1 or 2 times, which makes this avoidance outcome a count variable rather than a continuous variable. A logistic link function for count data was used. Via a binomial distribution the number of avoidance responses out of a pre-specified number of possibilities (i.e., a maximum of two) was modeled [31]. This probability of avoidance was fitted by a hierarchical regression that made use of a logistic-binomial link function. Fig 5 displays this probability of avoidance over the stimulus dimension for both training conditions.

Extending the random intercept model with a random linear stimulus dimension reached a better fit, *χ*^*2*^ (2) **=** 16.46, *p* < .01, an additional random effect of the quadratic stimulus dimension did not contribute to a better fit, *χ*^*2*^ (3) **=** 6.24, *p* = .10. The parameter estimates of this model can be found in Table 1 and were in line with our hypothesis: results showed that both training groups have a linear increase in the probability of avoiding a stimulus that lies closer to the CS+, but that only in the relevant training group a significant quadratic slope effect of the stimulus dimension was present, compared to the group that received the irrelevant training.

## Discussion

Anxiety psychopathology has been associated with increased fear responses and avoidance towards ambiguous stimuli compared to healthy controls [5,6,16]. Since this increased fear generalization may play a role in the development or possibly further exacerbation of pathological fear, it is of clinical relevance to examine whether fear generalization can be reduced by promoting stimulus discrimination. In this study it was tested whether a perceptual discrimination training would result in less fear overgeneralization in a perceptual conditioning task compared to an irrelevant control training in a non-clinical sample.

The results show that the groups did not differ in fear generalization before training. After the training phase the group that received the relevant discrimination training was less likely to avoid ambiguous, generalized stimuli compared to the irrelevant control group, despite the absence of group differences on harm expectancies related to the generalized stimuli.

This is one of the first studies that attempts to test whether the promotion of discrimination ability could decrease fear generalization, by isolating this specific intervention used in clinical practice and bringing it to the lab. Although this study focused specifically on perceptual discrimination, it can be seen as a proof of principle and future studies could test the effect of discrimination training on other levels on which fear generalization occurs. Although group differences were only found on the behavioural outcome measure (avoidance) and not on the cognitive outcome measure (harm expectancy), the results can be seen as promising, given that avoidance of generalized stimuli maintains fear by preventing disconfirmation of erroneous fear beliefs. These results encourage further research on the effects of discrimination training on fear generalization in at-risk population, for example individuals high on neuroticism [16] or clinical populations, using a longer and more intense discrimination training.

A few limitations should be taken into account. First, the pre-training test did not show any group differences, but did not include a behavioural outcome measure, so no firm conclusions about causal effects of the training on avoidance behaviour can be drawn from this study. Possibly other factors like individual differences might have contributed to the group differences in avoidance of generalized stimuli, however, since there were no group differences on trait anxiety, neuroticism or worry it is unlikely that these variables that have been related to fear conditioning in earlier studies account for the difference in avoidance in the present study. With regard to the design, two CS+s and CS-s were chosen to increase the idea of a continuum instead of three categories: white (CS+/-), black (CS+/-) and all grey colours (GSs). Even though the colour differences for all stimuli were equal in terms of RGB values, it might have been easier to discriminate between pitch black and off-black and white and off-white than between grey colours. However, by using 2 CS+s and CS-s participants may learn in the acquisition phase that small colour differences are irrelevant. Even though the curve of US expectancies in the generalization phase before the training phase does not seem to support this and the relevant training group would still learn in the training phase that small differences are relevant, using only the off-white and off-black one as CS+ or CS- would be preferable in future studies. Furthermore, the relevant training only decreased avoidance of generalised stimuli, whereas harm expectancies were not affected. Possibly cognitive changes require more experiences that violate the harm expectancies in order to adapt them. Since all generalized stimuli were only shown twice in the generalization phase, this might have not been sufficient to result in harm expectancies changes. The discrepancy between training effects on US expectancy and avoidance encourage the inclusion of other outcome measures like psychophysiological measures that could provide further inside in which levels of fear generalization are affected by the discrimination training.

This study includes one of the first attempts to tackle the problematic fear overgeneralization as observed in clinical groups suffering from pathological anxiety. Together with results of a recent study in which a perceptual discrimination training task related to reduced fear generalization based on self-reported risk ratings of generalized stimuli [17], these results show promising evidence for the potential benefits of a discrimination training to diminish fear overgeneralization. It should be noted however, that the relationship between perceptual discrimination and fear generalization remains to be clarified by including concurrent recordings of subjective perception and fear responses to generalized stimuli [32]. Reduced perceptual discrimination could underlie fear overgeneralization, but generalization differences might also occur despite differences in discrimination ability. More insight in various mechanisms that could underlie gear overgeneralization is relevant, as these might require different clinical interventions.

In sum, in this study discrimination training was associated with less avoidance of ambiguous or generalized stimuli. Since avoidance behaviour maintains a sense of current threat [18] and prevents exposure to corrective experiences, interventions that promote stimulus discrimination may contribute to the treatment of anxiety related disorders such as PTSD.

## Supporting information

S1 File Acquisition phase pre-training select25_75. (CSV)

S2 File Acquisition phase post-training select25_75. (CSV)

S3 File Generalization phase pre-training select25_75. (CSV)

S4 File Generalization phase post-training selection 25_75 USexpectancy CS-s GSs CS+s. (CSV)

S5 File Generalization phase post-training selection 25_75 avoidance CS-s and GSs. (CSV)

S6 File Value names and descriptions excel files S1–S5. (DOCX)
