# Supplementary material for: Training discrimination diminishes maladaptive avoidance of innocuous stimuli in a fear conditioning paradigm
Source: PLoS One. 2017 Oct 12;12(10):e0184485. doi: 10.1371/journal.pone.0184485 (PMC5638232; doi:10.1371/journal.pone.0184485)
Supplement: S6 File — (DOCX) [file pone.0184485.s006.docx]

**S1 File** Excel file “acquisition phase pre-training select25_75” includes data of the acquisition phase before the training phase, only including those who learned the CS-US contingency

(value followed by the description of the value)

pp - participant number

training - 0=irrelevant training group; 1=relevant training group

UsexpCSMtr1 -US expectancy CS- acquisition phase trial 1

UsexpCSMtr2- US expectancy CS- acquisition phase trial 2

UsexpCSMtr3- US expectancy CS- acquisition phase trial 3

UsexpCSMtr4- US expectancy CS- acquisition phase trial 4

UsexpCSMtr5- US expectancy CS- acquisition phase trial 5

UsexpCSMtr6- US expectancy CS- acquisition phase trial 6

UsexpCSMtr7- US expectancy CS- acquisition phase trial 7

UsexpCSMtr8- US expectancy CS- acquisition phase trial 8

UsexpCSPtr1- US expectancy CS+ acquisition phase trial 1

UsexpCSPtr2- US expectancy CS+ acquisition phase trial 2

UsexpCSPtr3- US expectancy CS+ acquisition phase trial 3

UsexpCSPtr4- US expectancy CS+ acquisition phase trial 4

UsexpCSPtr5- US expectancy CS+ acquisition phase trial 5

UsexpCSPtr6- US expectancy CS+ acquisition phase trial 6

UsexpCSPtr7- US expectancy CS+ acquisition phase trial 7

UsexpCSPtr8- US expectancy CS+ acquisition phase trial 8

**S2 File** Excel file “acquisition phase post-training select25_75” includes data of the acquisition phase after the training phase, only including those who learned the CS-US contingency

(value followed by the description of the value)

pp: participant number

training: 0=irrelevant training group, 1=relevant training group

UsexpCSMtr1: US expectancy CS- acquisition phase trial 1

UsexpCSMtr2: US expectancy CS- acquisition phase trial 2

UsexpCSMtr3: US expectancy CS- acquisition phase trial 3

UsexpCSMtr4: US expectancy CS- acquisition phase trial 4

UsexpCSMtr5: US expectancy CS- acquisition phase trial 5

UsexpCSMtr6: US expectancy CS- acquisition phase trial 6

UsexpCSMtr7: US expectancy CS- acquisition phase trial 7

UsexpCSMtr8: US expectancy CS- acquisition phase trial 8

UsexpCSPtr1: US expectancy CS+ acquisition phase trial 1

UsexpCSPtr2: US expectancy CS+ acquisition phase trial 2

UsexpCSPtr3: US expectancy CS+ acquisition phase trial 3

UsexpCSPtr4: US expectancy CS+ acquisition phase trial 4

UsexpCSPtr5: US expectancy CS+ acquisition phase trial 5

UsexpCSPtr6: US expectancy CS+ acquisition phase trial 6

UsexpCSPtr7: US expectancy CS+ acquisition phase trial 7

UsexpCSPtr8: US expectancy CS+ acquisition phase trial 8

**S3 File** Excel file “generalization phase pre-training select25_75” includes data of the generalization phase before the training phase, only including those who learned the CS-US contingency

(value followed by the description of the value)

pp: participant number

training; 0=irrelevant training group, 1=relevant training group

Usexpcol1: US expectancy CS- 1 generalization phase pre-training

USexpcol2: US expectancy CS- 2 generalization phase pre-training

Usexpcol3: US expectancy GS1 generalization phase pre-training

USexpcol4: US expectancy GS2 generalization phase pre-training

USexpcol5: US expectancy GS3 generalization phase pre-training

USexpcol6: US expectancy GS4 generalization phase pre-training

USexpcol7: US expectancy GS5 generalization phase pre-training

USexpcol8: US expectancy GS6 generalization phase pre-training

USexp_CSM: US expectancy mean of two CS-1 and CS-2 generalization phase pre-training

**S4 File** Excel file “generalization phase post-training selection 25_75 USexpectancy CS-s GSs CS+s” includes data of the generalization phase after the training phase, only including those who learned the CS-US contingency, including the US-expectancy outcome measure data

(value followed by the description of the value)

pp: participant number

training: 0=irrelevant training group, 1=relevant training group

Usexpcol1: US expectancy CS-1 generalization phase post-training

USexpcol2: US expectancy CS-2 generalization phase post-training

Usexpcol3: US expectancy GS1 generalization phase post-training

USexpcol4: US expectancy GS2 generalization phase post-training

USexpcol5: US expectancy GS3 generalization phase post-training

USexpcol6: US expectancy GS4 generalization phase post-training

USexpcol7: US expectancy GS5 generalization phase post-training

USexpcol8: US expectancy GS6 generalization phase post-training

Usexpcol9: US expectancy CS+1 generalization phase post-training

Usexpcol10: US expectancy CS+2 generalization phase post-training

**S5 File** Excel file “generalization phase post-training selection 25_75 avoidance CS-s and GSs” includes data of the generalization phase after the training phase, only including those who learned the CS-US contingency, including the avoidance outcome measure data

(value followed by the description of the value)

pp: particpant number

training: 0=irrelevant training group, 1=relevant training group

noavoidedcol1: number of avoided CS-1 generalization phase post-training

noavoidedcol2: number of avoided CS-2 generalization phase post-training

noavoidedcol3: number of avoided GS1 generalization phase post-training

noavoidedno4: number of avoided GS2 generalization phase post-training

noavoidedcol5: number of avoided GS3 generalization phase post-training

noavoidedcol6: number of avoided GS4 generalization phase post-training

noavoidedcol7: number of avoided GS5 generalization phase post-training

noavoidedcol8: number of avoided GS6 generalization phase post-training
